# Dual Mechanisms of Metabolism and Gene Expression of the CCRF-CEM Leukemia Cells under Glucocorticoid Treatment

**DOI:** 10.3390/ijms22115889

**Published:** 2021-05-31

**Authors:** George I. Lambrou, Theodoros Karakonstantakis, Spiros Vlahopoulos, Apostolos Zaravinos

**Affiliations:** 1 Choremeio Research Laboratory, First Department of Pediatrics, National and Kapodistrian University of Athens, Thivon & Levadeias 8, Goudi, 11527 Athens, Greece; sblachop@med.uoa.gr; 2 Biochemical Department, “Aghia Sofia” Children’s Hospital, Thivon 1, Goudi, 11527 Athens, Greece; karakwn@yahoo.gr; 3 Department of Life Sciences, School of Sciences, European University Cyprus, 1516 Nicosia, Cyprus

**Keywords:** prednisolone, flow cytometry, proliferation, differential gene expression, apoptosis, invasion, microarray, CCRF-CEM

## Abstract

Background: Glucocorticoids play an essential part in anti-leukemic therapies, but resistance is a crucial event for the prognosis of the disease. Glucocorticoids influence the metabolic properties of leukemic cells. The inherent plasticity of clinically evolving cancer cells justifies the characterization of drug-induced early oncogenic pathways, which represent a likely source of detrimental secondary effects. Aim: The present work aims to investigate the effect of glucocorticoids in metabolic pathways in the CCRF-CEM leukemic cells. Metabolic factors and gene expression profiles were examined in order to unravel the possible mechanisms of the CCRF-CEM leukemic cell growth dynamics. Methods: CCRF-CEM cells were used as a model. Cells were treated with prednisolone with concentrations 0–700 μM. Cell culture supernatants were used for glucose, lactic acid, LDH, Na^+^, K^+^ and Ca^++^ measurements. Cytotoxicity was determined with flow cytometry. Microarray analysis was performed using two different chips of 1.2 k and 4.8 k genes. Gene Ontology enrichment analysis was applied to find metabolism- and GC-related genes. Results: Higher prednisolone concentrations inhibited glucose uptake, without exhibiting any cytotoxic effects. Glucose consumption did not correlate with the total cell population, or the viable population, indicating that growth is not directly proportional to glucose consumption. Neither of the subpopulations, i.e., viable, necrotic, or apoptotic cells, contributed to this. Conclusions: Different types of leukemic cells seem to exhibit different patterns of glucose metabolism. Both resistant and sensitive CCRF-CEM cells followed the aerobic pathway of glycolysis. There is probably a rapid change in membrane permeability, causing a general shutdown towards everything that is outside the cell. This could in part also explain the observed resistance. Glucocorticoids do not enter the cell passively anymore and therefore no effects are observed. Based on our observations, ion concentrations are measurable factors both in vitro and in vivo, which makes them possible markers of glucocorticoid cytotoxic action.

## 1. Introduction

### 1.1. Glucocorticoids and Apoptosis Resistance

Glucocorticoids (GC) are a significant therapeutic choice against acute lymphoblastic leukemia. However, resistance to GCs is a critical aspect in the prognosis of the disease [1,2]. We have previously shown that GC-resistant T-cell leukemia cells exhibit a biphasic mechanism of action of glucocorticoids [3]. However, it remains to be elucidated whether acute lymphoblastic leukemia cells have inherent mechanisms that induce GC tolerance, or whether they develop resistance responding to treatment [4,5]. Prednisolone is an important GC used to treat childhood acute lymphoblastic leukemia (ALL) [6]. The exact network of pathways activated by the glucocorticoid receptor (GR) is not completely understood, especially the mechanism by which GR stimulates apoptosis. The key defects present in GC-resistant cells have not yet been unambiguously characterized. Several explanations have been proposed on why leukemic cells exhibit resistance to prednisolone. One of these is somatic mutations in the GR gene, leading to inappropriate pathway activation or deactivation of the receptor itself [7,8]. The explanation of genetic variation in the GR gene has been contradictory since it seemed that several polymorphisms have been found in the normal population with glucocorticoid altered response [9]. Furthermore, corticosteroid resistance in childhood ALL cannot be attributed to an inability of resistant cells to up-regulate the expression of the GR upon steroid exposure, nor to differences in inducible usage of the GR promoter [10,11]. On the other hand, it is proposed that when genetic variation is present, especially in in vitro systems such as a cell line, then resistance is taken for granted, although there are cell lines that do not possess a somatic mutation and yet do manifest resistance to glucocorticoids [12,13]. One such case is the T-cell leukemia cell line that we used in the present study (CCRF-CEM), which has a L753F GR gene mutation on one allele that has already occurred in vivo and impairs ligand binding [14,15]. CCRF-CEM cells possessing this mutation seem to express a mutated GR that has equal trans-repression capabilities, but need 200-fold concentrations of glucocorticoid for transactivation. Nevertheless, AP-1 trans-repression can take place in the absence of a ligand [16,17].

There is a debate on whether in vitro or ex vivo systems are the best models to use for this type of study [18]. Both models approach the reality of the human body, but provide answers to the same topic, from different perspectives, each having advantages and disadvantages. On the one hand, in vitro systems do not represent reality as far as disease state is concerned, and the cells used in such systems are largely modified in order to be immortalized. On the other hand, the use of ex vivo systems is not experimentally solely sufficient, since it will be difficult to identify the pivotal regulatory signals that cause resistance to chemotherapy in patients, as locating and studying the cells affected at the exact time point of the initial regulatory change is impossible. The in vitro systems offer reproducibility and the opportunity to cross-examine intracellular signals by interfering with the pathways involved. They also provide subsequent analysis of the gene clusters regulated by a specific stimulus such as prednisolone.

### 1.2. Tumor Metabolomics

The field of metabolic mechanics of tumors, and in particular, the metabolic mechanics of acute lymphoblastic leukemia, is still largely unknown. It is possible that an answer to the treatment of cancer lies within the delicate mechanisms of metabolic processes. Warburg saw that cancer cells change their metabolism from oxidative phosphorylation to aerobic glycolysis (the *Warburg effect*) [19].

The idea of interfering with the metabolic pathways in cancer cells, as a therapeutic option, seems to be of great importance [20]. This became apparent from discoveries made in the field of metabolomics, involving molecules that were previously thought to be solely metabolic and without the slightest suspicion for regulatory functions. Such molecules include diacylglycerol [21], ceramides and sphingosine, molecules essential in cell proliferation and apoptosis [22], as well as pyridine nucleotides, with an essential role in signal transduction [23]. It is clear nowadays that metabolites or metabolic molecules not only participate in metabolic processes which relate solely to the energy production and thermodynamical conservation of the cell, but also possess multiple functions, especially at the level of signal transduction. Therefore, it would not be an exaggeration to say that the same molecules used as a nutrient could also be the therapeutic targets.

### 1.3. GC-Induced Apoptosis Resistance and High Throughput Methodologies

Elucidation and better understanding of the action of glucocorticoids will allow us to identify the responsible gene targets for glucocorticoid resistance. This may lead to the discovery of drugs, such as inhibitors of overexpressed genes, which, in combination with glucocorticoids, might increase the effectiveness of anti-leukemia therapies [24]. The CCRF-CEM cells that were used in this study are from a GC-resistant cell line that probably resulted from the gathering of more resistant variants after long culturing periods [25,26]. In this study, the observed resistance behavior was consistent with previous studies as well as a more recent reports for dexamethasone-resistant CCRF-CEM cells [27]. These cells are not clonally homogeneous, but that is also the case with patients in vivo.

The use of high-throughput technologies, such as microarrays, has provided new insights into the diagnosis, prognosis and treatment response of acute lymphoblastic leukemia to new therapeutic targets. Further on, glucocorticoids are a natural mechanism for the control of lymphocyte clone growth [28,29,30], a mechanism that also has a role in limiting the growth of malignant lymphocyte clones [31]. These facts make the study of GCs a very important aspect, since any progress towards the issue of resistance to GC-induced apoptosis could prove crucial for acute lymphoblastic leukemia therapy.

### 1.4. Scope of the Present Work

The present work aims to investigate the mechanics of leukemic cell proliferation under prednisolone treatment. We measured the physical parameters of the system under investigation, including cell growth, consumption rate and its time derivative of the observed processes. This is the first effort to model the effects of prednisolone on a T-cell leukemia cell line.

## 2. Results

CCRF-CEM cells were treated with seven different, clinically relevant concentrations to determine the cytotoxic effect of prednisolone as a chemotherapeutic agent in vitro, and to study its effects on the distribution of the cell cycle and the expression of cancer-specific genes at an early and late response.

### 2.1. Cell Proliferation, Growht Rate, Growth Acceleration

**Figure 1:**
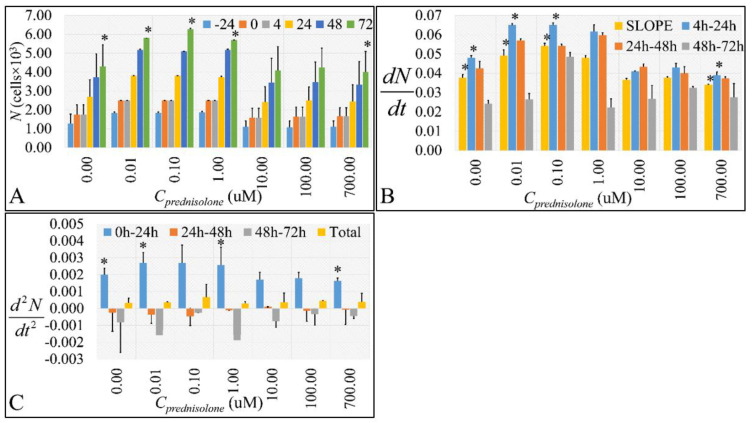
The effect of prednisolone on cell population as detected by the *Coulter* method. We estimated the absolute cell population (**A**), along with its change rate (*dN/dt*) (**B**) and cell size change acceleration (*d*^2^*N/dt*^2^) (**C**). Cell population change rate was estimated as the first derivative of cell population in each time point. Cell population change acceleration was calculated as the second derivative of the cell population in each time point (* depict a significance at the *p* < 0.01 level. “Total” implies the change rate and change rate acceleration respectively from time point 0 h to 72 h).

Cell proliferation was assessed at different time intervals to obtain an initial evaluation of prednisolone. The doubling time of the cultured cells was 45.2 ± 5.5 h. Low prednisolone concentrations (10 and 100 nM) significantly increased cell population at 72 h (Figure 1A and Supplementary Figure S1A). On the other hand, the population growth for untreated cells remained very close to the growth observed at concentrations higher than 10 μM (Figure 1A and Supplementary Figure S1A). It was evident that cells were resistant to prednisolone. At the same time, the proliferation rate (*dN/dt*) manifested significant differences. In particular, the decisive step in cell growth rate was the transition point from 4 to 24 h, where the low concentrations manifested the higher “velocity” of proliferation. At almost all concentrations, the proliferation rate was reduced at 72 h, which was a sign of reversal in the cell’s tendency to divide further (Figure 1B and Supplementary Figure S1B). This was also confirmed by our calculation of the “acceleration” or “deceleration” of cell growth (*d*^2^*N/dt*^2^) (Figure 1B and Supplementary Figure S1B). The decisive step was the transition from time 0 to 24 h, where cells manifested an “explosion” in terms of accelerating. However, in the next time points, a deceleration is observed. When accounting for the total effect (Figure 1C and Supplementary Figure S1C “Total”) of prednisolone from time point 0 to 72 h, cells were growing with an increasing “speed”, a fact that hints towards a metabolic challenge for cell homeostasis (Figure 1C and Supplementary Figure S1C).

### 2.2. Cell Biological Properties

#### 2.2.1. Cell Size

**Figure 2:**
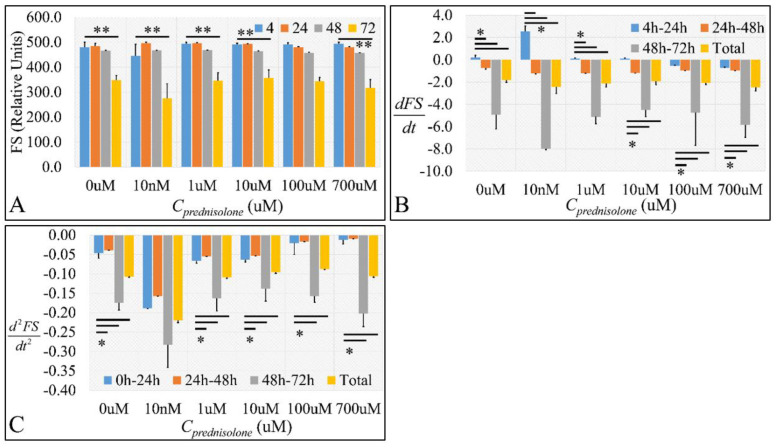
The effect of prednisolone on cell size as detected by flow cytometry. We estimated the absolute cell size (**A**), along with cell size change rate (**B**) and cell size change acceleration (**C**). Cell size change rate was estimated as the first derivative (*dFS*/*dt*) of cell size in each time point and cell size change acceleration was calculated as the second derivative (*d*^2^*FS*/*dt*^2^) of the cell size in each time point (** depicts a significance at the *p* < 0.001 level between concentrations and * depicts a significance at the *p* < 0.01 level. “Total” implies the change rate and change rate acceleration respectively from time point 0 h to 72 h).

Cell size was also assessed at different time intervals to obtain an initial evaluation of prednisolone using the forward scatter (FS) output from flow cytometry. In all cases, cell size was significantly decreased at 72 h, compared to 4 h of treatment (Figure 2A and Supplementary Figure S2A). Interestingly, all prednisolone concentrations manifested comparable cell size decrease at 72 h, indicating that cell size changes were independent of drug treatment (Figure 2A and Supplementary Figure S2A). On the other hand, the population’s cell size change rate (*dFS/dt*) also manifested significant differences, between the rate at 24 h and all other time-points. Interestingly, cell size manifested a tendency of increase initially, which changed at the next time-points (Figure 2B and Supplementary Figure S2B). Similarly, this was also confirmed by our calculation of the “acceleration” or “deceleration” of cell size (Figure 2C and Supplementary Figure S2C).

#### 2.2.2. The Discrimination of Cell Populations Based on Prednisolone’s Cytotoxicity

**Figure 3:**
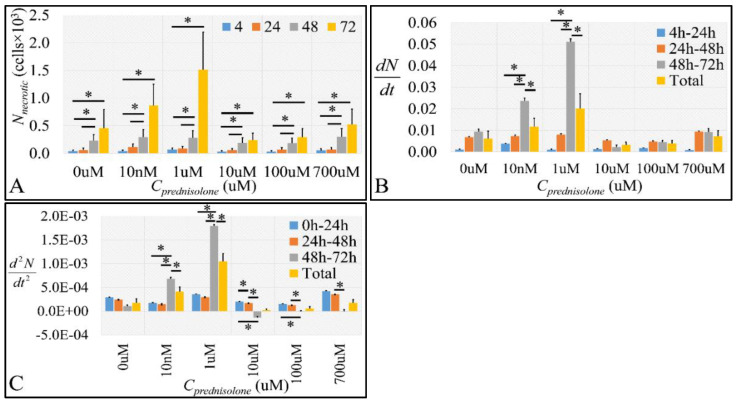
The effect of prednisolone on cell necrosis as detected by flow cytometry. We estimated the absolute necrotic cell population (**A**), along with the necrotic cell population change rate (**B**) and necrotic cell population change rate “acceleration (**C**). Necrotic cell population change rate was estimated as the first derivative (*dN/dt*) of necrotic cell population in each time point and cell size change “acceleration” was calculated as the second derivative (*d*^2^*N*/*dt*^2^) of the necrotic cell population in each time point (* depicts a significance at the *p* < 0.01 level between concentrations. “Total” implies the change rate and change rate acceleration respectively from time point 0 h to 72 h).

Prednisolone exposure for 4, 24, and 48 h did not have a significant effect on GC-induced necrosis (Figure 3A and Supplementary Figure S3A). Necrosis demonstrated a maxima at 72 h for the 1 μM prednisolone concentration, while at the highest prednisolone concentration (700 μM), necrosis was comparable to that observed for untreated cells (Figure 3A). It appeared that prednisolone manifested a biphasic effect for cell necrosis as well, which was probably caused by the increased metabolic needs of leukemic cells, as cells treated with 1 μM prednisolone manifested higher growth levels compared to the control (Figure 1A). However, this was not observed at the 10 nM prednisolone concentration, enhancing the dose-dependent biphasic prednisolone effect. Metabolic challenge could also be suggested by the finding that necrosis advances with increasing “velocity” (Figure 3B and Supplementary Figure S3B) and “acceleration” (Figure 3C and Supplementary Figure S3C).

**Figure 4:**
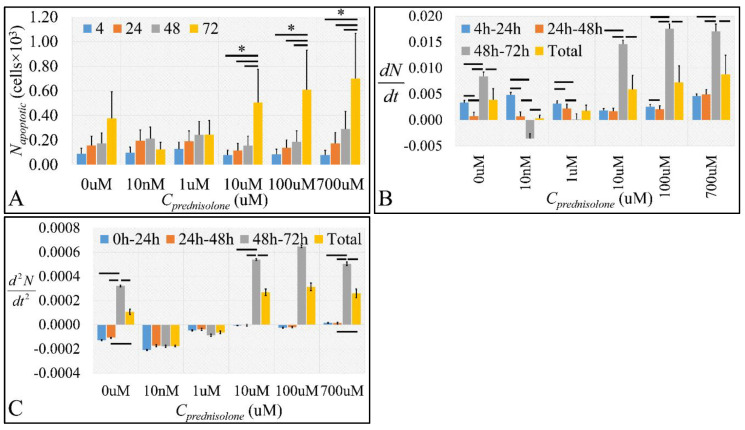
The effect of prednisolone on cell apoptosis as detected by flow cytometry. We estimated the absolute apoptotic cell population (**A**), along with the apoptotic cell population change rate (**B**) and apoptotic cell population change rate “acceleration” (**C**). Apoptotic cell population change rate was estimated as the first derivative (*dN/dt*) of apoptotic cell population in each time point and cell size change “acceleration” was calculated as the second derivative (*d*^2^*N/dt*^2^) of the apoptotic cell population in each time point (* depicts a significance at the *p* < 0.01 level between concentrations. “Total” implies the change rate and change rate acceleration respectively from time point 0 h to 72 h).

It appears that 1 μM is a threshold prednisolone concentration for the dose-dependent biphasic effect. Both “velocity” and “acceleration” increase up to 1 μM, fall close to control levels, to rise again in a dose-dependent manner. This can also suggest that at low doses, prednisolone has an immediate effect by causing “sudden death” in the cells, probably because of high receptor binding affinity and a gradual effect leading to apoptosis, perhaps because of receptor desensitization. Necrosis manifested a maxima at 72 h for the 1 μM prednisolone concentration, while necrosis at the highest prednisolone concentration (700 μM) was comparable to that observed for untreated cells (Figure 4A and Supplementary Figure S4A). It appeared that prednisolone manifested a biphasic effect for cell necrosis as well, which was probably caused by the increased metabolic needs of leukemic cells, as cells treated with 1 μM prednisolone manifested higher growth levels compared to the control (Figure 1A). However, this was not observed at the 10 nM prednisolone concentration, enhancing the dose-dependent biphasic prednisolone effect. Metabolic challenge could also probably be suggested by the finding that necrosis advances with increasing “velocity” (Figure 3B and Supplementary Figure S3B) and “acceleration” (Figure 3C and Supplementary Figure S3C). It appears that 1 μM is a threshold prednisolone concentration for the dose-dependent biphasic effect. Both “velocity” and “acceleration” increase up to 1 μM, fall close to control levels, to rise again in a dose-dependent manner. This can also suggest that at low doses, prednisolone has an immediate effect by causing “sudden death” in the cells, probably because of high receptor binding affinity and a gradual effect leading to apoptosis, probably because of receptor desensitization.

As in the case of necrotic cell population, apoptosis was more evident at 72 h of prednisolone treatment. An interesting finding was that the 1 μM concentration consisted of a “threshold”, where smaller concentrations manifested an anti-apoptotic effect and higher concentrations produced an apoptotic effect (Figure 4A and Supplementary Figure S4A). This biphasic effect was also manifested in the calculation of apoptosis “velocity” (Figure 4B and Supplementary Figure S4B) and “acceleration” (Figure 4C and Supplementary Figure S4C). Both physical quantities manifested a change after the 1 μM concentration and 72 h of prednisolone treatment.

Comparing the two forms of cell death, after 72 h, there was a significant apoptotic and necrotic effect with a “preference” to necrosis at low doses (10 nM, 1 μM, *p* < 0.05) (Figure 3A) and apoptosis at high doses (10, 100, and 700 μM, *p* < 0.01) (Figure 4A). The threshold concentration for this “preference” seemed to be the 1 μΜ. Above 1 μM prednisolone, there was an increase in both necrosis and apoptosis with increasing prednisolone concentrations, and a sudden shift from necrosis to apoptosis somewhere between 10 and 700 μM. Interestingly, prednisolone treatment at very low doses (10 nM, 1 μM) resulted in reduced apoptosis compared to the untreated cells (Figure 4A, *p* < 0.05).

### 2.3. Cell Metabolic Properties

**Figure 5:**
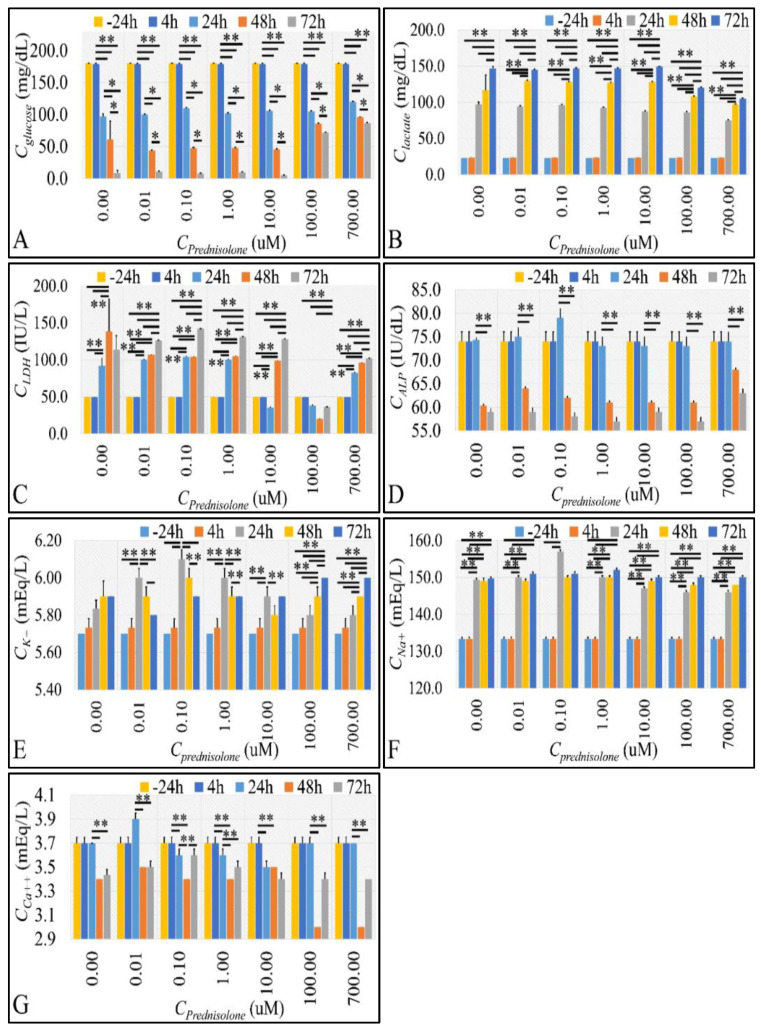
The effect of prednisolone cell metabolic factors. We measured the concentrations of glucose (**A**), lactate (**B**), lactate dehydrogenase (LDH) (**C**), alkaline phosphatase (ALP) (**D**), potassium (*K*^+^) (**E**), sodium (*Na*^+^) (**F**) and calcium (*Ca*^+2^) (**G**), with respect to both prednisolone concentration and time (** depict a significance at the *p* < 0.01 level between time-points and * depicts a significance at the *p* < 0.01 level. “Total” implies the change rate and change rate acceleration respectively from time point 0 h to 72 h).

Increased proliferation was accompanied by glucose consumption comparable to control levels (Figure 5A), whereas higher concentrations manifested a decreased glucose consumption as compared to control samples and lower concentrations (Figure 5A). At the same time, lactate followed the exact opposite dynamics, which was expected if cells were to follow the anaerobic glycolysis pattern (Figure 5B). Lactate dehydrogenase (LDH) increased in a time-dependent manner (Figure 5C), and at the same time, followed the necrosis dynamics observed previously (Figure 3A). Alkaline phosphatase (ALP) followed a similar path as LDH (Figure 5D). Serum ions also manifested an interesting behavior. Potassium levels manifested a maxima at 24 h, for prednisolone concentrations up to 10 μM and a time-dependent increase for higher prednisolone concentrations (Figure 5E). Similar behavior was observed for sodium ions (Figure 5F). Interestingly, potassium ions manifested significant positive correlation with total cell population at all prednisolone concentrations (*ρ* = 0.88, *p* = 0.04 at 4 h, *ρ* = 0.87, *p* = 0.05 at 24 h, *ρ* = 0.90, *p* = 0.03 at 48 h and *ρ* = 0.88, *p* = 0.04 at 72 h). Calcium, on the other hand, followed ALP patterns, with the exception of higher prednisolone concentrations at 72 h, which manifested an increase in calcium levels (Figure 5G).

### 2.4. Glucose Consumption Follows the Reaction Kinetics of Anaerobic Glycolysis

We initially observed a decrease in glucose uptake from lower concentrations to higher concentrations, with the 10 μM concentration being the limit of this change. This was observed from the measurement of glucose (Figure 5A) and its metabolite, lactic acid (Figure 5B).

We then questioned whether the consumption of glucose relates to the production of lactic acid. We hypothesized that the majority of glucose will turn into lactic acid and the rest will enter, either oxidative phosphorylation in the mitochondria or the pentose-phosphate pathway. If this is the case, the concentrations of glucose and lactic acid should correlate linearly. Indeed, when estimating the Pearson’s correlation coefficients, we found that glucose concentration was significantly anti-correlated to lactate concentration between 24 and 48 h (*ρ* = −0.92, *p* = 0.023), 24 h and 72 h (*ρ* = −0.87, *p* = 0.05) and 48 h and 72 h (*ρ* = −0.97, *p* = 0.003).

The transformation of glucose into two lactic acid molecules during anaerobic glycolysis (C_6_H_12_O_6_→2CH_3_CHOHCOOH + 2ATP, Equation (1)) can be written as:

C_6_H_12_O_6_ + 2NAD^+^ + 2ADP + 2P_i_→2H_2_O + 2CH_3_COCO_2_^−^ + 2NADH + 2ATP + 2H_3_O^+^ (1)

This is a lump reaction, representing the algebraic sum of many others. Assuming that there is not significant biochemical cross-talk of intermediate reactions with other external metabolic pools, the lumping of the reactions to a single one can be done. We measured the substrates of this reaction and included an estimate of its efficiency using Equation (3):

(2) $$\alpha =\frac{C_{observed}}{C_{theoretical}}$$

where *α* is the efficiency of the reaction and 0 < *α* < 1, *C_observed_* is the observed/measured yield of the reaction and *C_theoretical_* is the expected reaction yield if dealing with a perfect reaction. Since we are dealing with thermodynamical systems, the efficiency of the reactions is essential for the understanding of such systems. In reality, biological systems are far from thermodynamic equilibrium, therefore they are dissipative. In other words, they exchange matter and energy from their environment.

**Figure 6:**
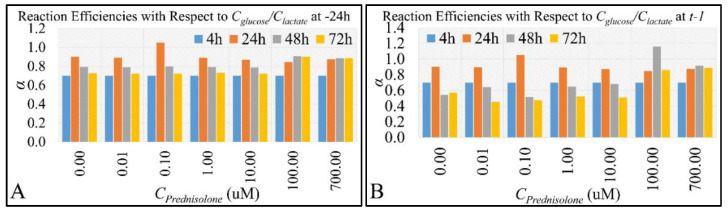
Reaction efficiencies (as depicted in Equation (1)) of glucose and its metabolite lactate. Reaction efficiencies were calculated both in a time- and dose-dependent manner. Efficiencies were calculated with respect to the mean of metabolites concentration at time –24 h and all prednisolone concentrations (**A**), as well as with respect to the metabolites concentrations at time *t* − 1, i.e., between *C_glucose_* and *C_lactate_* at time *t* and *C_glucose_* and *C_lactate_* at time *t* − 1 or each separate concentration (**B**).

We estimated *α* for all reactions in a dose- and time-dependent manner. As expected, we observed the highest reaction efficiencies at 24 h, which was previously shown to be a crucial step in cell growth “velocity” (Figure 1B). This was true for both efficiencies calculated with respect to the concentrations at −24 h (Figure 6A) as well as previous metabolite concentrations at time *t* − 1 (Figure 6B).

### 2.5. Correlations

**Figure 7:**
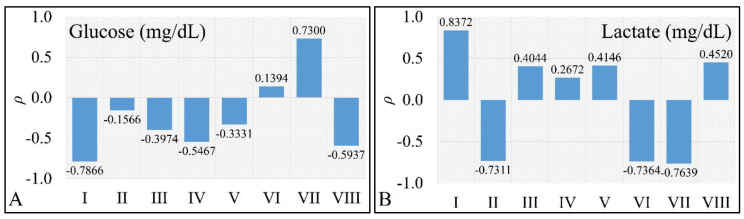
Pearson’s correlation of glucose (*C_Glucose_*) concentration with respect to other physical parameters (**A**) and lactate (*C_Lactate_*) concentration to other physical parameters (**B**). Each bar (depicted here as Latin numbers) represents the calculation of Pearson’s correlation of a pair of factors as described in detail in Table 1.

As previously postulated, if there was a correlative pattern in the effects of prednisolone treatment on leukemic cells, it would be reasonable to expect certain patterns of correlation. However, due to the observed biphasic effect of the drug, we should expect no significant correlations among measured and calculated physical values. Our assumption proved correct since we did not find significant correlations between estimated values (Figure 7). In particular, we would expect to find positive or negative correlations between glucose concentration and cell populations, as well as between lactate concentration and cell populations. Interestingly, cell size (as indirectly measured with flow cytometry) did not manifest significant correlations (*ρ* = −0.09) at 72 h with respect to glucose concentrations, while it manifested significant correlations (*ρ* > 0.8) for all concentrations with respect to time. In addition, no significant correlation was found for cell size and the apoptotic or necrotic populations.

### 2.6. Regressions

**Figure 8:**
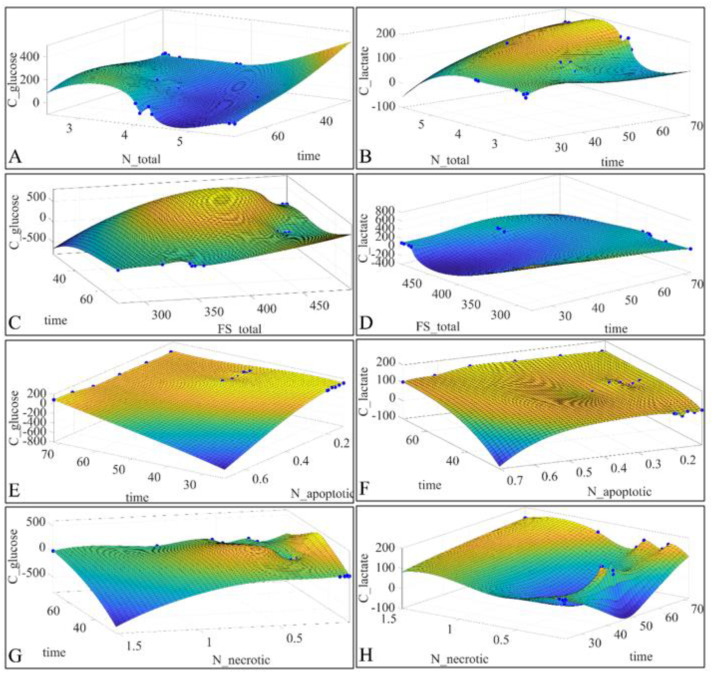
Surface regressions of the parameters for which Pearson’s correlations were calculated. Plotted variables included the *C_Glucose_* vs. the total cell population (*N*) and vs. time (**A**), the *C_Lactate_* vs. the total population (*N*) and vs. time (**B**), the *C_Glucose_* vs. the flow cytometric forward scatter (FS) and vs. time (**C**), the *C_Lactate_* vs. the flow cytometric forward scatter (FS) and vs. time (**D**), *C_Glucose_* vs. the apoptotic cell population (*N_Apoptotic_*) and vs. time (**E**), the *C_Lactate_* vs. the apoptotic cell population (*N_Apoptotic_*) and vs. time (**F**), the *C_Glucose_* vs. the necrotic cell population and vs. time (**G**) and finally the *C_Lactate_* vs. the necrotic cell population and vs. time (**H**).

It became evident that correlations of the evaluated parameters, including both the cytological data as well as gene expression data, were not of a linear nature and thus it is reasonable that such correlations are of non-linear nature. In order to depict the inter-relations between the estimated variables for which we had previously calculated the Pearson’s correlation factor, we plotted 3D surface plots of the parameters under investigation. In particular, we plotted the *C_Glucose_* vs. the total cell population (*N*) and vs. time (Figure 8A), the *C_Lactate_* vs. the total population (*N*) and vs. time (Figure 8B), the *C_Glucose_* vs. the flow cytometric forward scatter (FS) and vs. time (Figure 8C), the *C_Lactate_* vs. the flow cytometric forward scatter (FS) and vs. time (Figure 8D), *C_Glucose_* vs. the apoptotic cell population (*N_Apoptotic_*) and vs. time (Figure 8E), the *C_Lactate_* vs. the apoptotic cell population (*N_Apoptotic_*) and vs. time (Figure 8F), the *C_Glucose_* vs. the necrotic cell population and vs. time (Figure 8G) and finally the *C_Lactate_* vs. the necrotic cell population and vs. time (Figure 8H). All surface plots were regressed with an interpolant, biharmonic approximation manifesting their non-linear dynamics.

### 2.7. Gene Expression

#### 2.7.1. Hierarchical Clustering (HCL)

Prednisolone exerted late cytotoxic effects on the cell line, which we hypothesized could be reflected on the cell’s expression profile. Thus, we used microarrays focusing on the early response of differential gene expression profiles. We chose to focus on early response, and specifically on the GR/NF-κB-linked, direct target genes, to diminish the complexities that arise later due to ensuing feedback mechanisms. The time points that we chose were expected to manifest the first reaction of cells to prednisolone as well as give an image of its late effects. Based on the flow cytometry and cell proliferation findings, the gene expression profiles were analyzed after 4 h of exposure to prednisolone (when the immediate transcriptional response to prednisolone exposure should be expected) as well as after 72 h of exposure to it. We chose the concentrations of 10 nM and 700 μM as they comprised the minima and maxima of prednisolone concentrations, corresponding to the extreme effects that we observed by flow cytometry, i.e., anti-apoptosis accompanied with mitogenic effect and cytotoxicity accompanied with resistance. Moreover, we used the high concentration (700 μM), similar to concentrations used in different studies in both CCRF-CEM cells [27] as well as primary cell lines derived from pediatric patients [18]. Out of 1.2 k transcripts, 24 were found to be differentially expressed, including *CFLAR*, *CXCL8*, *DAD1*, *ENC1*, *ENDOU*, *EPHB6*, *EVPL*, *FLT1*, *FOS*, *HPRT1*, *MMP1*, *NPAT*, *OSTF1*, *PCSK7*, *PDCD10*, *RUBCNL*, *SEMA3C*, *SERPINB2*, *SOD1*, *TGFB1*, *TGFBR2*, *TPBG*, *UMPS* and *XRCC6*.

**Figure 9:**
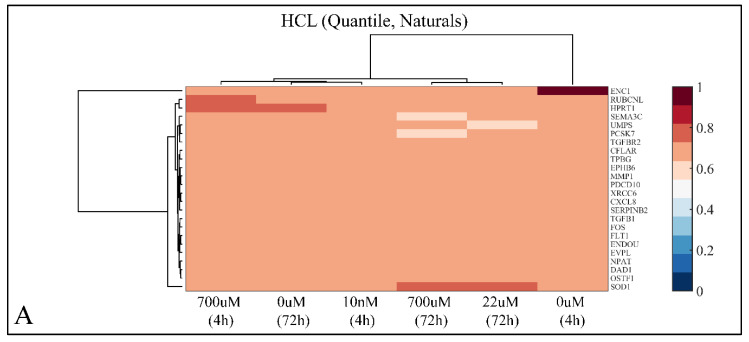

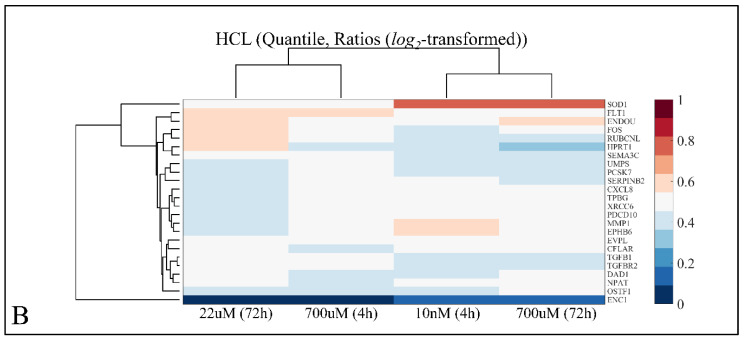
Hierarchical clustering (HCL) analysis of gene expression. Gene expression was clustered with respect to the natural values (normalized, DE, not *log*_2_-transformed genes) (**A**), as well as with respect to the ratios (normalized, DE, not *log*_2_-transformed genes) (**B**).

Genes were clustered both with respect to their natural values (gene expression values that were normalized, identified as DE and not *log*_2_-transformed), as well as with respect to their ratio values (*log*_2_-transformed). Based on our previous observations, if gene expression is to follow the biphasic effect of prednisolone on the leukemic cells, samples should not be clustered together with respect to their expressional profile. This was the case, as hierarchical clustering (HCL) showed that 0 μM at 72 h clustered together with 10 nM at 4 h and they both clustered together with 700 μM at 4 h (Figure 9A). In addition, 700 μM at 72 h clustered together with 22 μM at 72 h and 0 μM at 4 h was clustered independently (Figure 9A). Regarding the ratios of gene expression, 22 μM at 72 h and 700 μM at 4 h where clustered together, and 10 nM at 4 h clustered together with 700 μM at 72 h (Figure 9B).

#### 2.7.2. K-Means Clustering

We then used k-means clustering to identify possible genes that function in a biphasic pattern as observed in the treatment of prednisolone. In other words, we would expect to find genes that manifest different patterns of expression, that are either up- or down-regulated or with very low and very high expression values (and vice versa), respectively. Differentially expressed genes (DEGs) displayed four clusters for both the natural values of gene expression (Supplementary Figure S5A,B), as well as for gene expression ratios (Supplementary Figure S5C,D). Interestingly, cluster 1 (Supplementary Figure S5A) revealed that *SOD1* manifested low expression compared to high prednisolone concentration. Cluster 4 (Supplementary Figure S5A) manifested a set of genes (*DAD1*, *EPHAB6*, *ENDOU*, *FLT1*, *FOS*, *HPRT1*, *NPAT*, *OSTF1* and *RUBCNL*) that followed the growth pattern of prednisolone treated cells, i.e., expression was elevated at 700 μM compared to 10 nM prednisolone treatment. At the same time, gene ratios revealed a third cluster (*ENC1*) (Supplementary Figure S5C), manifesting a similar effect, i.e., high expression value at 10 nM compared to 700 μM at 4 h and raised again at 700 μM at 72 h.

#### 2.7.3. Gene Functional Analysis

**Figure 10:**
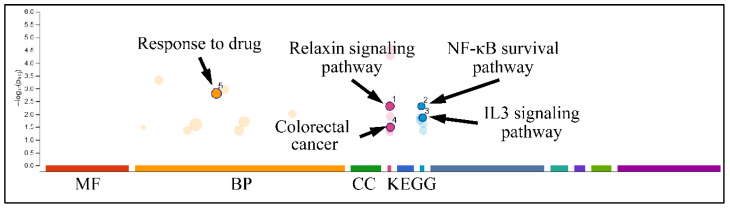
Gene Ontology (GO) analysis. DEGs were analyzed for their known functions using gprofiler (Legend: MF: Molecular Function, BP: Biological Process, CC: Cellular Component, KEGG: KEGG pathway database).

**Figure 11:**
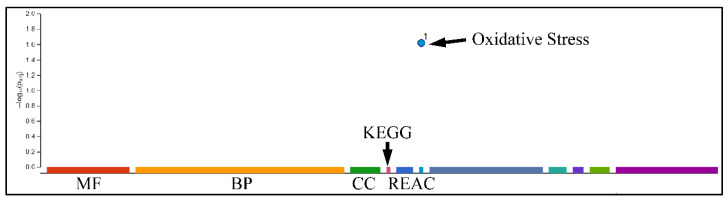
Gene Ontology (GO) analysis. Differentially expressed genes (DEGs) were analyzed for their known functions using gprofiler (Legend: MF: Molecular Function, BP: Biological Process, CC: Cellular Component, KEGG: KEGG pathway database, REAC: Reactome database).

Following this, we performed functional analysis to identify relative GC functions in the genes identified by k-means clustering. DEGs manifested several interesting functions, with respect to the experimental setup, where they appeared to participate in response to drug functions, the relaxin signaling pathway, colorectal cancer, the NF-κB survival pathway and IL3 signaling pathway (Figure 10). Interestingly, oxidative stress was significantly enriched when analyzing the genes revealed in k-means analysis (Figure 11).

## 3. Discussion

In the present work, cells that were treated with low to medium prednisolone doses exhibited higher proliferation rates compared to the controls, while, on the other hand, higher doses of prednisolone had similar proliferation dynamics compared to the controls. At the same time, glucose measurements showed the control and low to medium prednisolone doses manifested a rapid consumption of glucose within 72 h, while higher doses manifested a much slower consumption. However, the proliferation potential did not change between untreated cells and those treated with high doses of prednisolone. This formed a paradox, since higher concentrations manifested a growth rate and population similar to that of untreated cells. It would be expected that prednisolone would not affect glucose uptake and that the cells’ energetics are independent from the mechanisms exerted by the glucocorticoid receptor. This observation leads us to the conclusion that prednisolone affected the mechanism of glucose uptake, and interestingly the same effect was reported a long time ago by Lippman et al. (1974) [25].

To give a possible answer to our observations, we hypothesized the following: either high doses of prednisolone altered the membrane’s permeability and uptake of glucose, or prednisolone obstructs the glucose receptors. A third hypothesis is that prednisolone reverses the Warburg effect in leukemic cells and shifts it towards autophagy.

The case of no change due to membrane permeability should be ruled out because of cell growth, appeared to be positively correlated with *K+* approximately at <24 h after treatment initiation. Interestingly, *Na+* appeared to be positively correlated with cell population only at 72 h of treatment, indicating a feedback mechanism of *Na+* regulation. Further on, HCL of gene expression did not group the experiments between them, indicating that genes are in an interplay for proliferation and survival.

A strong relation between tumor progression and metabolism, i.e., glucose uptake and impaired metabolic pathways, has been well documented [32,33,34]. Yet, since solid tumor metabolism is relatively well-documented, only a few studies exist on leukemia [35,36].

We also studied physical factors, such as the “velocity” and the “acceleration” within a biological system, and questioned whether they can be observed at the phenotypical level, and how this translates at the level of gene expression. For example, an increase in the proliferation rate of a cellular population should mean that the required genes for this effect should have a faster transcription rate. Interestingly, Mar et al. (2009) suggested that gene expression takes place in quanta, i.e., it happens discretely and not continuously [37]. Furthermore, gene expression was reported to follow oscillatory patterns, complicating things even further [38]. This means that cells cannot simply transit from one state to the other as they grow. If the hypothesis of oscillatory patterns in gene expression is true, a much more complicated regulatory pattern is required by a cell to change its state, reacting to an environmental stimulus. In the present work, we provide evidence supporting this view, regarding glucocorticoids. We emphasized changes in cell populations during treatment with glucocorticoids, in a spatio-temporal manner. Previous works dealt with this issue, emphasizing the glucocorticoid receptor and the pharmacokinetics of glucocorticoids (methylprednisolone) [39,40].

Further on, we investigated the changes on cell size, which is a topic which has not received much attention in the literature. We observed that cell size reduced with respect to time, but not with respect to concentration, and therefore we hypothesized that this change was not related to the effect of prednisolone. At the same time, the fact that no significant correlations were found with respect to the apoptotic or necrotic cell populations meant that we also hypothesized that cell death was not related to cell size, as it is expected that apoptotic cells cause cell size reduction. Therefore, it is possible that cell size reduction took place due to a secondary proliferative mechanism. Interestingly, this has been reported in an older study, for solid tissues and tumors [41], which is mainly due to the limitations in space. However, in the case of leukemia, space is not an imminent problem (especially in the case of a cell culture environment) and therefore, it is possible that cell shape alterations take place due to proliferative reasons. Noteworthy, previous reports linked the prognosis of leukemia with leukemic clone cell size with correlating larger cells to better treatment outcome [42,43].

The significance of our findings relies on setting up a modeling framework to describe the dynamics of leukemic cells under the influence of glucocorticoids. We used two factors in our analysis: cell populations, including changes in viability and cell death, and metabolic factors. As previously reported, glucose metabolism did not correlate with GC resistance in CEM cells [44] and at the same time it appeared that sensitive cells consumed glucose much faster than resistant cells. At the same time, another study suggested that glycolysis is upregulated in prednisolone resistance cells (Jurkat and Molt-4) [35,45]. These two findings imply that different types of leukemic cells have different patterns of glucose metabolism. The interesting thing is that in the case of CEM cells, irrespective of whether they were resistant or sensitive, they both followed the aerobic pathway of glycolysis producing almost exclusively lactic acid from glucose.

### 3.1. The Duality of Prednisolone Action

The present findings suggest a dual response capacity of the assayed ALL cell line to corticosteroid: activation of signals for proliferation and necrosis at low drug concentrations, along with the activation of an acute pathway for cell death (e.g., Caspase cascade) and apoptosis, as well as transcriptional and translational-dependent apoptotic pathways at high drug concentrations. One of the intermediate drug concentrations assayed (1 μM) caused the sharpest rise in G2-phase, accompanied with the lowest S/G1 ratio, and the highest fraction of necrotic cells as reported in a previous report [3]. On the other hand, the increase in prednisolone dose caused cells to accumulate at the G1 phase and led them to apoptosis.

The high levels of proliferation that we observed are in contrast with the total cell death at low concentrations. Even at high prednisolone concentrations, the proliferation rates were similar to those observed in untreated cells. A possible explanation for these observations is that prednisolone may exhibit three different constitutive effects in this model—specifically, mitogenic (proliferative), anti-apoptotic and cytotoxic. In addition, these behaviors are expressed not necessarily individually, but in combinations, as well. Within the 10 nM to 1 μM range, prednisolone acts both as a mitogen and anti-apoptotic agent. Apoptotic death is at lower levels than that of untreated cells. The peak in total cell death at 1 μM of prednisolone concentration is mainly an effect of a peak in necrosis for the same concentration. Despite the increased necrosis, cell growth exceeds that corresponding to the zero and higher doses due to the mitogenic effect. At higher doses (>1 μM), prednisolone functions as a dose-dependent cytotoxic agent, but still proliferation remains at the same levels as that of untreated cells. The dose dependency of mitogenic behavior is similar to that reported in several in vitro and in vivo studies for different cell types [46].

This observed behavior indicates a mechanism of delayed escalatory, dose-dependent apoptosis for concentrations higher than 1 μM upon 72 h. A delay in the action of corticosteroids depending on the cell type and lasting from 2 to 24 h was previously reported [47,48]. Interestingly, in a different study concerning lactic acidosis, it was observed that *bcl-2* expression causes a delay in glucocorticoid-induced apoptosis protecting the CCRF-CEM cell line from dexamethasone. In addition, in the same study dexamethasone seemed to induce a biphasic mechanism in cell death via the loss of mitochondrial function (resulting in lactic acidosis) [49]. In this study, it appears that the delay of the prednisolone action lasts up to 72 h, and this is supported by previous reports [27,50]. The mechanism of prednisolone action delay explains the dose-dependent preference of necrosis to apoptosis and at the same time, it partly explains the higher proliferation rate at low concentrations.

### 3.2. The Duality of DEGs

GC-regulated genes begin to influence subsequent gene expression. To reduce the complexity due to feedback mechanisms affecting gene expression, we obtained a snapshot of the early direct effects on gene expression by complementary DNA (cDNA) microarray analysis upon 4 h of treatment. This analysis was expected to include key genetic regulators and initiators of further downstream pathways. We found several genes (i.e., *EPHAB6*, *SOD1*, *DAD1*, *ENDOU*, *FLT1*, *FOS*, *HPRT1*, *NPAT*, *OSTF1*, *RUBCNL* and *ENC1*) manifesting a possible dual functionality in our GC-treated ALL cells.

One of these genes was *EPHB6*. Ephrin receptors and their ligands, the ephrins, mediate numerous developmental processes, particularly in the nervous system. Ephrins are divided into the ephrin-A (EFNA) class, which are anchored to the membrane by a glycosylphosphatidylinositol linkage, and the ephrin-B (EFNB) class, which are transmembrane proteins. Their receptors are also divided into 2 groups, based on the similarity of their extracellular domain sequences and their affinities for binding their ligands. Ephrin receptors make up the largest subgroup of the receptor tyrosine kinase (RTK) family. The ephrin receptor encoded by *EPHB6* lacks the kinase activity of most receptor tyrosine kinases and binds to ephrin-B ligands. It has been reported that *EPHB6* is over-expressed in acute myeloid leukemia. In addition, it is involved in T-cell maturation, since it appears to be over-expressed in the thymus. Interestingly, in order for the tyrosine receptor to be active, cell–cell interaction is obligatory. In the present study, this speculates that T-cells exchange signals not only through autocrine mechanisms but by direct contact, especially after being stimulated by low doses of prednisolone. Another fact reported, and consistent with our results, is that EphB6 stimulates T-cells to proliferate. In our study, it seems that prednisolone played the stimulus role only at low doses [51].

SOD1 participates in the response to oxidative stress and metabolic stress [52]. DAD1 is an anti-apoptotic gene, which has been found to be up-regulated by low prednisolone concentrations and down-regulated by higher prednisolone concentrations in our study. There are not many reports known that link DAD1 to acute lymphoblastic leukemia progression and proliferation, except one that reported its anti-apoptotic role in leukemic cells [53]. Another interesting gene appeared to be *ENDOU*, which codes for an endoribonuclease. Two recent reports showed that it is involved in amino acid uptake and metabolism of leukemic cells, whose inhibition manifests anti-leukemic effects [54,55]. This was in agreement with our study, since *ENDOU* was up-regulated in all prednisolone treatments and all time-points. We also focused on *HPRT1*, since its mutations are considered to play a significant role in resistance to thiopurine-induced cell death, with its overexpression being a prognostic factor for thiopurine resistance [56]. Interestingly, *HPRT1* was found to be down-regulated in our study at all concentrations and all time-points tested. *OSTF1* is a gene known for his function in bone metabolism. There are no studies concerning its function in leukemia. However, a recent report showed that *OSTF1* participates in calcium regulation, which probably hints towards that function in our study. *RUBCNL* is a gene for which no prior knowledge exists for its role in leukemia. Nevertheless, a recent report highlighted this gene as a molecular “switch” for cellular homeostasis, between autophagy and glucose metabolism [57]. It is probable that *RUBCNL* acts as the same molecular “switch” in our cells, enhancing our previous hypothesis for a transition to autophagy by higher prednisolone concentrations, thus enhancing cell survival.

### 3.3. The Translation of Experimental Findigs to the Treatment of Leukemia

GCs are considered to be first-line drugs in the treatment of acute lymphoblastic leukemia [3,31]. Resistance to GC-induced apoptosis is a major issue in the treatment of acute lymphoblastic leukemia and especially childhood acute lymphoblastic leukemia [3,31,58]. Drug-resistant forms of leukemia are the major cause of fatality in children. Most studies are concerned with the effects of GCs on metabolic factors, as well as metabolism-related secondary pathologies due to the GC-treatment [59,60]. However, there are metabolic aspects related to the resistance, per se, to glucocorticoids. Interestingly, in a previous work, it was shown that glycolysis was up-regulated in GC-resistant CEM cells [61], in agreement with our study, which linked the resistance or sensitivity to the pathway of metabolism. Aside from those observations, recent advances have highlighted that the reprogramming of metabolic pathways are possible targets for cancer therapy. As mentioned previously, the Warburg effect is considered a metabolic redirection in cancer, which is tightly linked to tumor survival [62]. Thus, targeting tumor metabolic pathways is considered a probable chemotherapeutic approach towards their treatment [63,64,65]. Another previous study has shown that resistance to GCs is linked to an increased proliferation rate, again in agreement with our study [66]. The increased proliferation is probably not directly linked to therapy, yet the genes involved in the proliferative effect are a possible therapeutic target in acute lymphoblastic leukemia [66].

### 3.4. The Role of the Microenvironemt: In Vitro vs. In Vivo

Leukemic cells not only interact with the bone marrow microenvironment, but also modulate the micro-environmental signal transduction by stimulating activated T cells and stromal cells with the help of chemokines and chemokine receptors [67]. Lymphocytes express functional molecules and chemokine receptors that direct the chemotaxis of leukemic cells in vitro. Restriction of lymphocytes to a specific site depends on the continuous entrapment of adhesion molecules and activation through chemokine receptors. Cytokines and chemokines support clone extension by promoting hyper-function of anti-apoptotic genes. These facts indicate that leukemic cells circulate and focus on and towards specific micro-environmental nests (niches) [68].

The proliferation of leukemic lymphocytes depends on contact with other lymphocytes which can support the growth of leukemic cells and can prevent apoptosis due to the interaction with bone marrow stromal cells, promoting survival. Schulz et al. (2011) demonstrated that while leukemic cells can survive for a long time in vivo, they need help from stromal cells to survive in vitro [69]. During the last decade, it has become apparent that external signals from the leukemic microenvironment contribute significantly to the development of leukemia, and emphasis is placed on the study of the leukemic microenvironment and the existing cross-talk between cellular and molecular pathways. The importance of T-lymphocytes remains controversial in B-cell leukemia, as T-cells can enhance the leukemic clone but can also suppress it. Adhesion molecules such as integrins play a central role in the interactions between normal and malignant hematopoietic cells and bone marrow microenvironment. Adhesion of leukemic cells to bone marrow stromal cells and extracellular matrix is necessary for the survival of leukemic cells [70].

Various proteins, e.g., CREBBP or CBP, play an important role in the bone marrow microenvironment and contribute to the differentiation of stem and progenitor cells, acquiring the leukemic phenotype. CREB binding protein (CREBBP) is an important protein for the autonomous cellular regulation of hematopoiesis, including the stem cell [71]. For example, it is known that the bone marrow microenvironment is suppressive for the active cells of the immune system, therefore promoting the leukemic clone progression [72,73,74]. Regulatory T-cells have been identified for being used by leukemic cells to evade the immune response. In addition, the protective effects of the marrow achieve protection against cytotoxic drugs, allowing the development of cell resistance that imposes the need for the development of appropriate drugs and the microenvironment [72,73,74].

Despite the significant progress that has been made, the exact mechanisms of the microenvironment’s role in leukemia progression are still largely unknown. It is certain, however, that there is a difference between in vivo and in vitro experimentation, as the second can serve as an approximation of the first. Further studies are needed on the role of cytokines, chemokines and their receptors in the formation and support of the microenvironment. Inflammatory cells, fibroblasts, monocytes, macrophages, dendritic cells, basophils, and T-cells appear to contribute to the leukemic microenvironment consisting of a complex mixture for leukemia progression.

## 4. Materials and Methods

### 4.1. Cell Culture

The T-lymphoblastic leukemia cell line CCRF-CEM was used as the model, obtained from the European Collection of Cell Cultures (ECACC). Cells were grown in RPMI-1640 medium supplemented with 2 mM L-glutamine and streptomycin/penicillin 100 U/mL (Gibco), 20% FBS (Gibco) at 37 °C, 5% CO_2_ and ~100% humidity. Twenty-four hours before application of prednisolone (which is called –24 h time at the present study) cells were harvested by centrifugation at 1000 rpm for 10 min on a KUBOTA centrifuge and seeded at an initial concentration of 0.9 × 10^3^ to 1.3 × 10^3^ cells/μL in a final medium volume of 10 mL [3].

### 4.2. Prednisolone Treatment

Prednisolone (Pharmacia) of initial concentration 1 mg/mL was diluted to obtain the following different concentrations: as aforementioned, the average in vivo concentration was calculated to be ~100 μM. The remaining concentrations were calculated as follows 0 μM (control), 10 nM (3.90 × 10^−6^ mg/mL), 1.09 μM (3.946 × 10^−4^ mg/mL), 10 μM (3.946 × 10^−3^ mg/mL), 50 μM (1.58 × 10^−2^ mg/mL), 100 μM (3.16 × 10^−2^ mg/mL), 175 μM (6.31 × 10^−2^ mg/mL), and 700 μM (0.253 mg/mL). Cell number was determined at –24 h and the next day at 0 h, where prednisolone was administrated. Cell number was then determined at 4 h and subsequently every 24 h for a total time of 72 h.

### 4.3. Cell Proliferation

In order to determine the cell population a NIHON KOHDEN CellTaq-α hematology analyzer was used (sensitivity setting: 5, threshold: 2). Cells were counted at −24, 0, 4, 24, 48, and 72 h. For this purpose, 200 µL were taken from each flask and counted directly without any other treatment with the analyzer. Cell population doubling time (*t_d_*), in *h*, was calculated by:

(3) $$t_{d}={\left(\frac{\Delta N_{t}}{\Delta t}3.32\right)}^{-1}$$

### 4.4. RNA Extraction

RNA was isolated with Trizol (Thermo Fisher Scientific Inc., Waltham, MA, USA). After isolation, the RNA amount was measured with a spectrophotometer (*BioRad SmartSpec 3000*) and its integrity was tested with 2% agarose gel electrophoresis. At least 40 μg of RNA was used from each sample, i.e., control sample, 10 nM prednisolone and 701 μM prednisolone. Since there is no method that can yield DNA-free RNA, we followed DNase treatment (RQ1 DNAse, Promega) as described by the manufacturer. Finally, RNA samples were further cleaned with RNA-Easy Mini Kit (QIAGEN) and again the amount of RNA was determined through spectrophotometry as described above and RNA integrity was determined using agarose gel electrophoresis. Samples with a 1.8 to 2.0 ratio were kept and in addition, those that empirically showed the 28 S band being twice as bright as the 18 S band on the gel. The exact process was previously described in [3,5].

### 4.5. Flow Cytometry

Flow cytometry was performed on a *Beckman Coulter* flow cytometer FC500. Cytotoxicity was determined via AnnexinV-PI staining with the Vybrant Apoptosis Assay Kit #2 (Alexa Fluor 488 annexin V/Propidium Iodide) (Thermo Fisher Scientific Inc., Waltham, MA, USA) as described by the manufacturer and previously reported [3,4]. Briefly, 1 mL of cell suspension was taken from each flask, measured to determine the cell number and centrifuged. Supernatant was removed and cells were washed with 1 mL PBS, re-centrifuged at 1000 rpm for 10 min. Supernatant was removed and cells were suspended in 100 µL 1× Annexin Binding Buffer to obtain a final concentration of ~1 × 10^6^ cells. Cells were stained with AnnexinV/Alexa 488-PI incubated for 15 min in dark and 400 µL of 1× Annexin Binding buffer was added.

Cell cycle distribution and DNA content was determined with standard PI (propidium iodide, Thermo Fisher Scientific Inc., Waltham, MA, USA) staining as described elsewhere. Briefly, 1 mL was taken from each flask and centrifuged at 1000 rpm for 10 min. Supernatant was removed and cells were suspended in 1 mL 75% ethyl alcohol (EtOH). Cells were incubated overnight at 4 °C or for longer periods at −20 °C. After incubation cells were centrifuged at 1000 rpm for 10min. Supernatant was removed and cells were washed with 1 mL 1× PBS, pH 7.4. Cells were re-centrifuged and re-diluted in 1 mL 1× PBS pH 7.4. RNase A was added and cells were incubated at 37 °C for 30 min in order to remove any remaining traces of RNA that could interfere with PI. Ten microliters of PI of initial concentration 1 mg/mL was added to a final concentration of 1 ng/mL. All concentrations and time point experiments consist of triplicate experiments [3,5].

### 4.6. Biochemical Measurements

Supernatants from the cell culture were taken at 0 h and every 24 h thereafter and kept at −80 °C until further processing. In brief, 1 mL of cell culture media was taken, centrifuged at 1200 rpm for 10 min and the supernatant was removed and kept for further processing. Samples were then measured with a Siemens biochemical analyzer. Supernatants from the cell culture were taken every 24 h and kept at −80 °C thereafter until further processing. Samples were then measured with a Siemens biochemical analyzer *Advia 1800*. The factors measured were glucose (mg/dlt), lactic acid (mg/dlt), lactate dehydrogenase (LDH, IU/lt), alkaline phosphatase (ALP, IU/lt), K^+^ (mmole/lt), Na^+^ (mmole/lt), Ca^++^ (mmole/lt) and Mg^++^ (mmole/lt).

### 4.7. Microarray Experimentation

cDNA microarray chips were obtained from TAKARA the *Human Cancer Chip v.40* with 1.2 k transcripts [75,76] and the *IntelliGene*™ II Human CHIP 1, respectively, with 4.8 k transcripts [38]. Hybridization was performed with the CyScribe Post-Labeling kit (RPN5660, Amersham) using Cy3 and Cy5. cDNAs were purified with QIAGEN PCR product clean-up kit (Cat # 28104). Slides were activated at 55 °C for 30 min in 1% BSA. Samples were applied on the slides, and left to hybridize overnight at 55 °C. The following day, slides were washed in 200 mL 0.1 × SSC and 0.1% SDS for 3 × 5 min, in 200 mL 0.1 × SSC for 2 × 5 min and in 200 mL ddH_2_O for 30 s. Slides were dried by centrifugation at 1500 rpm for 3 min and scanned with a microarray scanner (ScanArray 4000 XL). Images were generated with Scan Array microarray acquisition software (GSI Lumonics Inc., Bedford, MA, USA). To obtain the highest possible experimental accuracy, cDNAs were used from three experimental setups, each one consisting of three independent experiments. In particular, the experimental setups consisted of the three following pairs for gene expression at 4 h: control (0 μM prednisolone) (Cy3) vs. 10 nM prednisolone (Cy5) (designated as 0 vs. 1), 10 nM prednisolone (Cy3) vs. 701 μM prednisolone (Cy5) (designated as 1 vs. 3), control (0 μM prednisolone) (Cy3) vs. 701 μM prednisolone (Cy5) (designated as 0 vs. 3). This is a “simple loop” experimental design, taking into account all possible combinations between samples, as previously described [77]. The sum of the ratios of the 0 vs. 1 and 1 vs. 3 experiments should theoretically equal the ratio in the 0 vs. 3 experiment. Experiments at 72 h were based on a simple reference design. In particular, 22 μM vs. 700 μM prednisolone at 72 h (designated as ‘4′), and control vs. 700 μM prednisolone at 72 h (designated as ‘5’). The 4 h experiments were performed in triplicate and the 72 h experiments in single experiments. Our design was described previously [3]. Raw data have been uploaded and are available at the GEO database with accession numbers GSE27989 to GSE28154.

### 4.8. Data Analysis

#### 4.8.1. Flow Cytometry Data Analysis

Flow cytometric data were analyzed with the Wi nMDI software for cytotoxicity data as well as for changing the file format to *.fcs. In addition, ploidy was determined with Wi nMDI and cell cycle distribution was determined with Cychlred. Flow cytometry and cell cycle data were analyzed with *Wi nMDI* software version 2.8 (*The Scripps Institute, Flow Cytometry Core Facility*) (http://facs.scripps.edu/software.html) and *Cylchred* version 1.0.2 (*Cardiff University, Wales*) (http://www.cardiff.ac.uk/medic/aboutus/departments/haematology/cytometry/cytonetuk/software/index.html), which is based on the algorithms proposed by Watson et al. and Ormerod et al. [78,79]. Statistical analysis was performed using the t-test for the proliferation, cytotoxic and cell cycle data. All cytometric data were considered statistically significant if they obtained a *p*-value of *p* < 0.05.

#### 4.8.2. Microarray Data Analysis

Microarray image acquisition was performed with the Scan Array microarray acquisition software (GSI Lumonics, Bedford, MA, USA) and raw data extraction was performed with the ImaGene v.6.0 Software (BioDiscovery Inc., El Segundo, CA, USA).

Data pre-processing was performed in Microsoft Excel^®^ and data processing was performed within the Matlab^®^ v.7.6.0 (The MathWorks Inc., Natick, MA, USA) computing environment, using the Bioinformatics Toolbox.

##### Background Correction

The well-performing multiplicative background correction (MBC) approach, as proposed by [80], was followed. MBC, which represents a combination of simplicity and efficient performance as regards background correction, assumes that the background noise affects the spot intensities in a multiplicative manner. Thus, instead of subtracting the background intensities from the foreground intensities, MBC subtracts the logarithmic estimates of the background intensities from the logarithmic foreground intensities as:

(4) $$F_{c}^{R,G}=F^{R,G}-B_{l}^{R,G}$$

where $F_{c}^{R,G}$ is the logarithmic background-corrected foreground intensity, and $B_{l}^{R,G}$ is the robust estimate of background noise, for each channel. The absolute background-corrected foreground intensity $F_{c}^{R,G}$ for each channel is then calculated as:

(5) $$F_{c}^{R,G}=2^{F_{c}^{R,G}}$$

To reduce the complexity of the dataset, we followed the replicate averaging approach proposed by [81], where the replicates were averaged by taking their geometric mean:

(6) $$F_{a}^{R,G}=\sqrt[{3}]{F_{r_{1}}^{R,G}\cdot F_{r_{2}}^{R,G}\cdot F_{r3}^{R,G}}$$

where $F_{r_{i}}^{R,G}$ is the (background-corrected) foreground intensity of the replicate *r_i_*, *i =* 1, 2, 3, and $F_{a}^{R,G}$ is the averaged foreground intensity across all replicates (henceforth referred to simply as signal intensity), for each channel (Red and Green).

##### Calculation of Experimental Means

The mean ratio of each triplicate of the *i*th gene was calculated as:

(7) $$R=\frac{1}{j}\sum_{i=1}^{j}\log_{2}\left(\frac{F_{r_{i}}^{R}}{F_{r_{i}}^{R}}\right)$$

where *R* is the mean expression ratio of a gene among a triplicate, and $F_{r_{i}}^{R}$ and $F_{r_{i}}^{G}$ are the normalized intensities of the two channels (Cy5 and Cy3 respectively). Equivalently stated, it is the geometric mean where:

(8) $$R=\log_{2}(GM)=\log\left(\sqrt[{j}]{\prod_{i=1}^{j}\frac{F_{r_{i}}^{R}}{F_{r_{i}}^{R}}}\right),j=3$$

##### Normalization

Normalization was performed using three algorithms; (a) the Loess algorithm with a quadratic polynomial model, and a smoothing parameter equal to 10%, which was considered appropriate for the relatively small number of probes attached in the microarrays, while (b) the Rank Invariant normalization algorithm was also applied and (c) the Quantile algorithm. The three algorithms were compared for their efficiencies. In general the Rank Invariant algorithm performed better as comparted to the other two.

##### Microarray Statistical Analysis

In order to identify potentially differentially expressed genes (DEG) between samples and among genes of the same experiment, we used Student’s *t*-test [82]. DEGs were identified by comparing all experiments of prednisolone treatment to all control samples at all time points. The reason for such comparison was that we were seeking those genes with the significant difference at all experimental stages, as these would be the ones (if any) that would affect cell behavior with respect to prednisolone. The false discovery rate (FDR) was calculated as described previously [83]. The DE genes per experiment were identified at a confidence level of 95%. Genes were considered to be significantly differentially expressed if they obtained a *p*-value < 0.05 and a FDR value < 0.05 in all experiments. The false discovery rate was calculated as described previously [83]. There was a FDR of 0.5% for a combined *p* < 0.05 for experimental setup 0 vs. 1, 0.6% for a combined *p* < 0.05 for experimental setup 1 vs. 3 and 0.9% for a combined *p* < 0.05 for the experimental setup 0 vs. 3.

##### Correlation Analysis

Correlation analysis was performed using Pearson’s correlation (r) and Spearman rank order correlation (ρ).

##### Unsupervised Classification Analysis

To gain further insight into the gene expression data, we used a set of unsupervised machine learning and classifying methods. These included hierarchical clustering (HCL) and k-means classification, recently reported as one of the best performing clustering approaches for microarray class discovery studies [84]. The k-means algorithm applied uses the squared Euclidean as a distance measure, since it is generally considered to be a more appropriate measure for use with k-means and found to outperform for ratio-based measurements. In addition, to prevent the local minimum problem, the algorithm was repeated for a number of runs with different initializations. Specifically, the clustering procedure was repeated 100 times, each with a new set of initial cluster centroid positions (seeds), selected at random, and the best run (the one that minimizes the sum, over all clusters, of the within-cluster sums of object-to-cluster-centroid distances) was taken as the final result. The implementation of all preprocessing and analysis steps, apart from the Gene Ontology based analysis that follows, took place within the Matlab^®^ 12 (R2016a) (The MathWorks Inc. Natick, MA, USA) computing environment. Optimal cluster number for the k-means algorithm, was estimated using the *Calinski-Harabasz* criterion. All methods were used in order to find patterns in gene expression with respect to the day/night cycle. Classifiers analyses were performed with the Matlab^®^ (The MathWorks, Inc. Natick, Boston) computational environment. In addition, a methodology of self-organizing maps (SOMs) was used in order to determine patterns of expression and it was compared to the hierarchical clustering results.

##### Gene Ontology (GO) Analysis

Gene Ontology (*GO*) analysis was performed using the gprofiler [85] and *WebGestalt* web-tools [86]. Relations of the differentially expressed genes and the transcription factor binding motifs were further investigated using the *Pubgene Ontology Database* (www.pubgene.org). Gene definitions and functions were based on the *National Institute of Health* databases (http://www.ncbi.nlm.nih.gov/sites/entrez/).

##### Pathway Analysis

Pathway analysis was performed using the gprofiler [85] and *WebGestalt* web-tools [86].

## 5. Conclusions

In the present work, we tested seven different, clinically relevant concentrations as a dose-dependent model and we studied the effect of prednisolone as a function of time. Interestingly, the proliferation rate of the cells was not affected by the concentration of prednisolone. Cytotoxicity assays revealed a high proportion of necrotic cells at the lowest prednisolone concentrations and, at the same time, very low apoptotic numbers. On the other hand, 1 μM seemed to be the threshold concentration for necrosis: as concentration rises, apoptotic death affects more cells than necrotic. Apoptosis was very low at 10 nM, rising from 10 μM and above. No dose-dependent cumulative cytotoxic effect of prednisolone was observed at 4 or 24 h. Only after 48 h of incubation was a dose-dependent escalation of cytotoxicity evident; cytotoxicity clearly manifests as a dose-dependent effect of corticosteroid stimulation at 72 h post-treatment. In conclusion, the present study provides an insight into the following aspects of prednisolone action: (a) induction of resistance on the parental CCRF-CEM cell line, (b) late cytotoxic effects on CCRF-CEM, indicating a delayed mechanism of action, (c) a dual mechanisms on cells, as far as apoptosis and necrosis are concerned, while synergism between mitogenic and anti-apoptotic effects antagonizes the effects of cytotoxic pathways on population size, (d) a dual mechanism of metabolism where cells reduce glucose uptake, while retaining growth at higher prednisolone concentrations and (e) the identification of several genes with a respective biphasic differential expression profile among low and high prednisolone doses reflected on its late effects. Such an approach allows the identification of genes that may constitute molecular targets for drugs in combination therapy with GCs. These drugs may affect the potential of GCs to inhibit growth of resistant acute lymphoblastic leukemia cells. This approach could also provide the potential early markers for GC resistance. GC resistance, as a prognostic factor, is evaluated from the cytotoxic effects on leukemic cells after a certain period of exposure to GCs. The early detection of resistance would give the possibility of averting such obstacles and making therapies more efficient.

## Figures and Tables

**Table 1:** Explanation of the Pearson’s correlation combinations used in Figure 7.

Latin Enumeration	Parameter 1	vs.	Parameter 2
I	$\frac{dC_{Glu\cos e\_or\_Lactate}}{dt}$	vs.	$\frac{dN}{dt}$
II	$\frac{d^{2}C_{Glu\cos e\_or\_Lactate}}{dt^{2}}$	vs.	$\frac{d^{2}N}{dt^{2}}$
III	$\frac{dC_{Glu\cos e\_or\_Lactate}}{dt}$	vs.	$\frac{dFS}{dt}$
IV	$\frac{d^{2}C_{Glu\cos e\_or\_Lactate}}{dt^{2}}$	vs.	$\frac{d^{2}FS}{dt^{2}}$
V	$\frac{dC_{Glu\cos e\_or\_Lactate}}{dt}$	vs.	$\frac{dN_{necrotic}}{dt}$
VI	$\frac{d^{2}C_{Glu\cos e\_or\_Lactate}}{dt^{2}}$	vs.	$\frac{d^{2}N_{necrotic}}{dt^{2}}$
VII	$\frac{dC_{Glu\cos e\_or\_Lactate}}{dt}$	vs.	$\frac{dN_{apoptotic}}{dt}$
VIII	$\frac{d^{2}C_{Glu\cos e\_or\_Lactate}}{dt^{2}}$	vs.	$\frac{d^{2}N_{apoptotic}}{dt^{2}}$
VII	$\frac{dC_{Glu\cos e}}{dt}$	vs.	$\frac{dN_{apoptotic}}{dt}$
VIII	$\frac{d^{2}C_{Glu\cos e}}{dt^{2}}$	vs.	$\frac{d^{2}N_{apoptotic}}{dt^{2}}$

## Supplementary Materials

Figures S1–S5 are available online at https://www.mdpi.com/article/10.3390/ijms22115889/s1.

## Abbreviations

Abbreviation	Explanation
ALL	Acute Lymphoblastic Leukemia
AML	Acute Myeloblastic Leukemia
BAX	BCL-2 associated X protein
BCL-2	B-cell CLL/lymphoma 2
BCR/ABL	breakpoint cluster region/v-abl Abelson murine leukemia viral oncogene homolog1
BFM trials	Berlin-Frankfurt-Muenster trials
DHFR	dihydrofolate reductase
DMSO	Dimethyl Sulfoxide
FDR	False Discovery Rate
FDR	False Discovery Rate
GC	Glucocorticoid
GM	Geometric Mean
GR	Glucocorticoid Receptor
GRE	Glucocorticoid Response Element
HCL	Hierarchical Clustering
IL-3	interleukin-3
LMO1	LIM domain only 1 (rhombotin 1)
LMO2	LIM domain only 2 (rhombotin-like 1)
LYL1	lymphoblastic leukemia derived sequence 1
MR	Mineralocorticoid Receptor
NF-κB	Nuclear factor of kappa in B-cells
N_t_	is the counted number of cells at time t_n_
N_t−1_	is the counted number of cells at time t_n−1_
PI	Propidium Iodide
RFC1	reduced folate carrier 1
SDS	Sodium Dodecyl Sulfate
SSC	Saline Sodium Citrate Buffer
TAL1	T-cell acute lymphocytic leukemia 1
TEL/AML1	alias for ETV6 (ets variant gene)/alias for CBFA2 (Core-binding factor alpha subunit 2; acute myeloid leukemia 1 gene; aml1 oncogene)
