# Supplementary material for: Dual Mechanisms of Metabolism and Gene Expression of the CCRF-CEM Leukemia Cells under Glucocorticoid Treatment
Source: Int J Mol Sci. 2021 May 31;22(11):5889. doi: 10.3390/ijms22115889 (PMC8198442; doi:10.3390/ijms22115889)
Supplement: Supplementary file 1 [file ijms-22-05889-s001.zip › ijms-933484-supplementary.pdf]

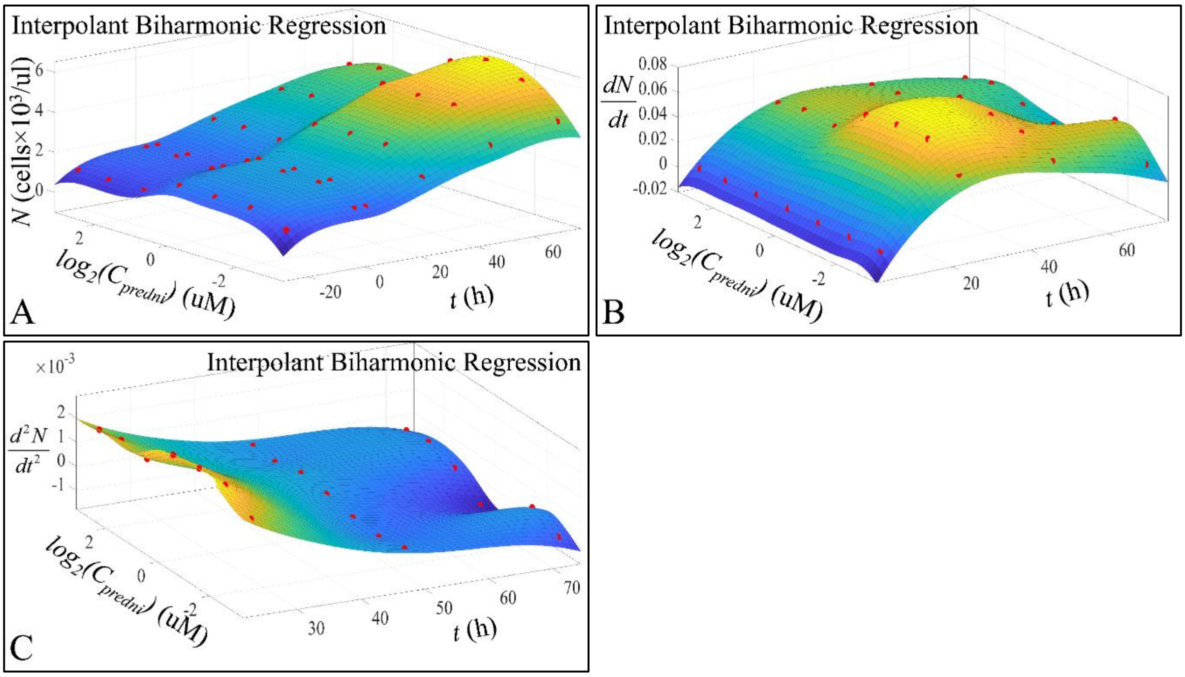

Figure S1. 3D regressions on the effect of prednisolone on cell population as detected by the *Coulter* method. We regressed the absolute cell population (**A**) as presented in **Fig. 1A**, along with its change rate (*dN*/*dt*) (**B**) as presented in **Fig. 1B** and cell size change acceleration (d^2N/dt^2) (**C**) as presented in **Fig. 1C**. Cell population change rate was estimated as the first derivative of cell population in each time point. Cell population change acceleration was calculated as the second derivative of the cell population in each time point.

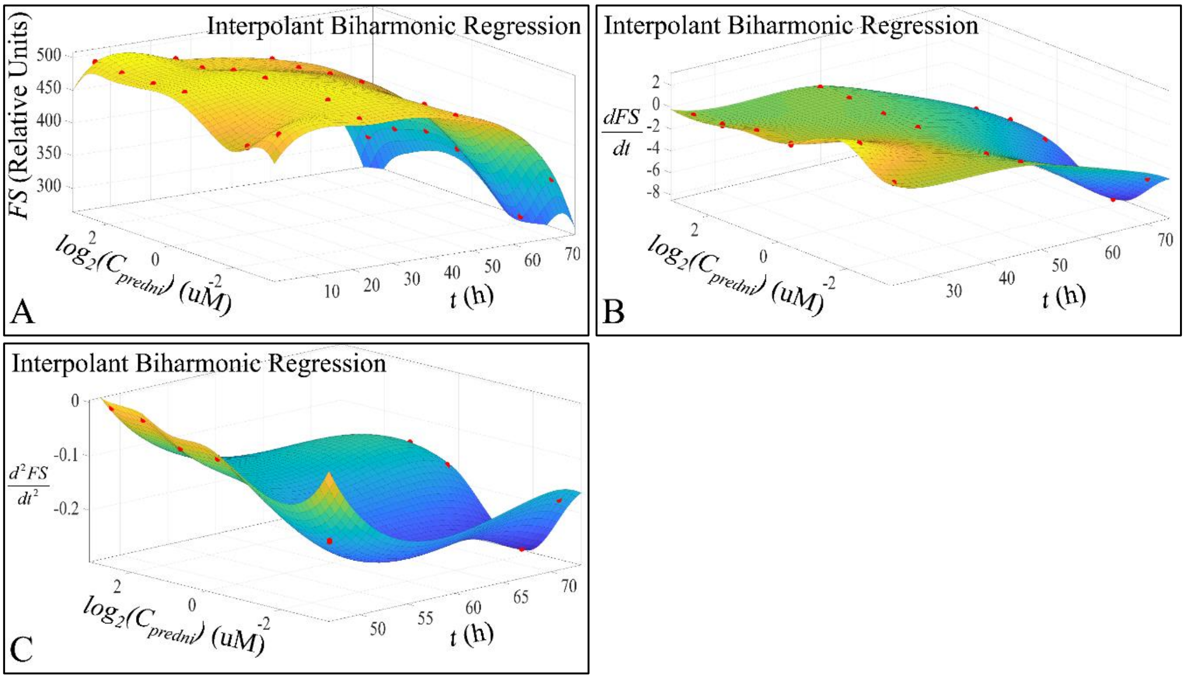

Figure S2. 3D regressions on the effect of prednisolone on cell size as detected by flow cytometry. We regressed the absolute cell size (**A**) as presented in **Fig. 2A**, along with cell size change rate (**B**) as presented in **Fig. 2B** and cell size change acceleration (**C**) as presented in **Fig. 2C**. Cell size change

rate was estimated as the first derivative (*dFS/dt*) of cell size in each time point and cell size change acceleration was calculated as the second derivative (d^2FS/dt^2) of the cell size in each time point (** depict a significance at the p<0.001 level between concentrations and * depicts a significance at the p<0.01 level. "**Total**" implies the change rate and change rate acceleration respectively from time point 0 h to 72 h).

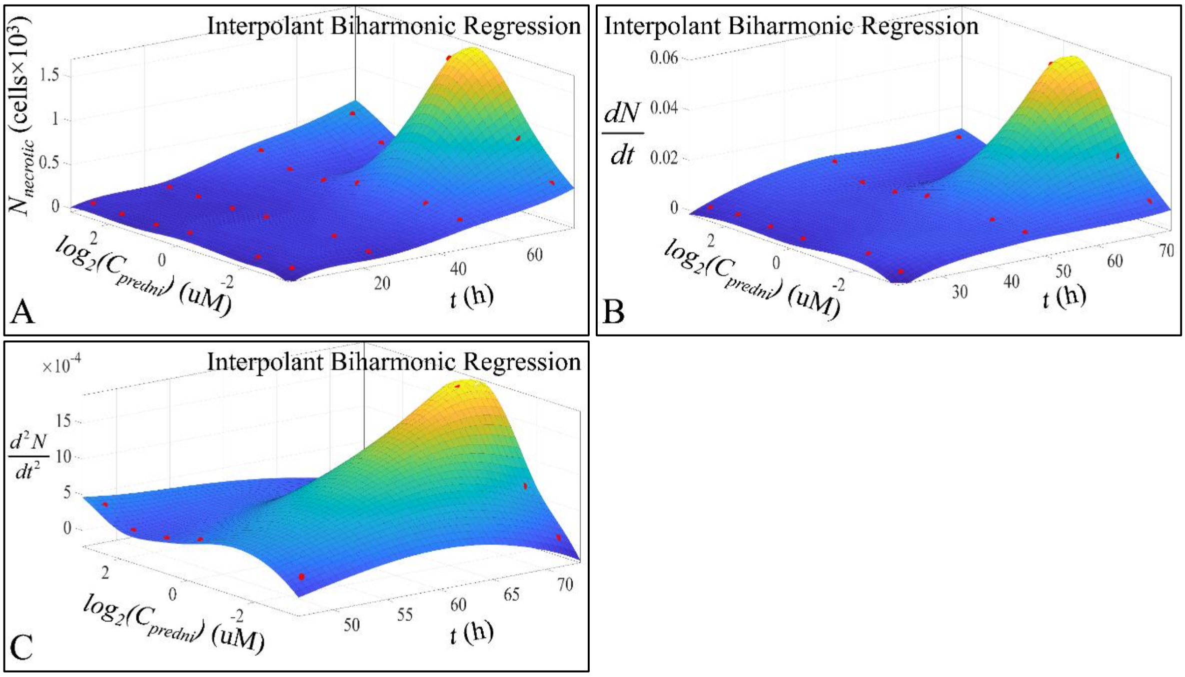

Figure S3. 3D regressions on the effect of prednisolone on cell necrosis as detected by flow cytometry. We regressed the absolute necrotic cell population (**A**) as presented in **Fig. 3A**, along with the necrotic cell population change rate (**B**) as presented in **Fig. 3A** and necrotic cell population change rate "acceleration: (**C**) as presented in **Fig. 3A**. Necrotic cell population change rate was estimated as the first derivative (*dN/dt*) of necrotic cell population in each time point and cell size change "acceleration" was calculated as the second derivative (d^2N/dt^2) of the necrotic cell population in each time point (* depict a significance at the p<0.01 level between concentrations. "**Total**" implies the change rate and change rate acceleration respectively from time point 0 h to 72 h).

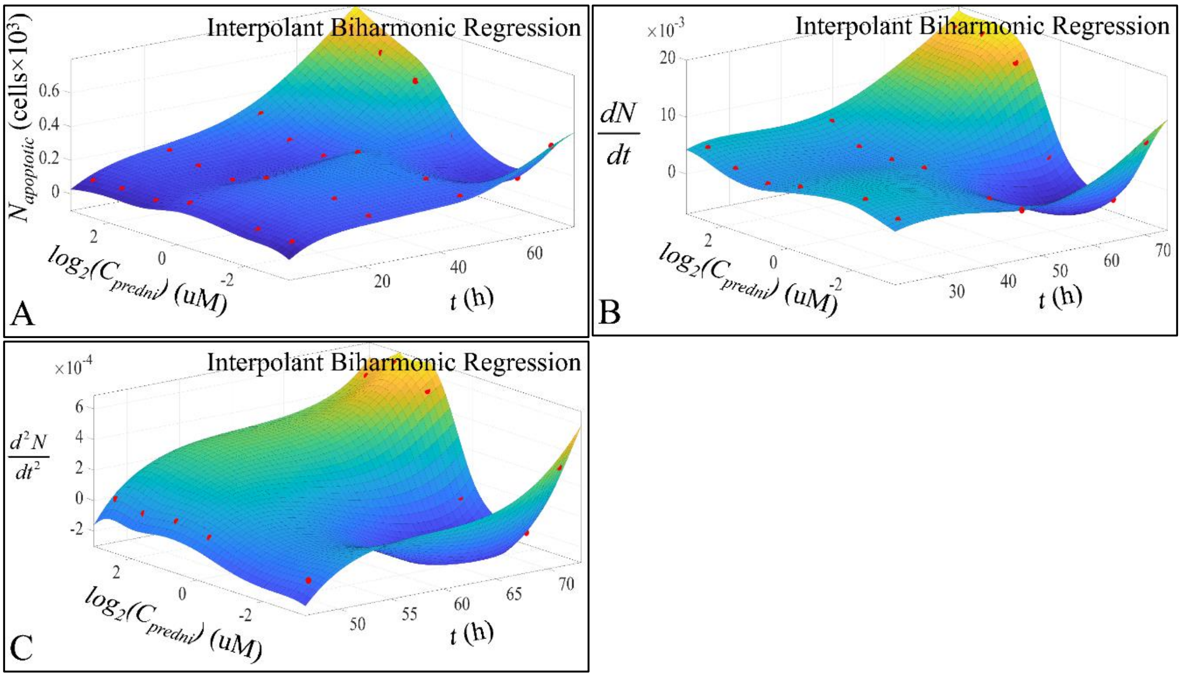

Figure S4. 3D regressions on the effect of prednisolone on cell apoptosis as detected by flow cytometry. We regressed the absolute apoptotic cell population (**A**), along with the apoptotic cell population change rate (**B**) and apoptotic cell population change rate "acceleration" (**C**). Apoptotic cell population change rate were estimated as the first derivative (*dN*/*dt*) of apoptotic cell population in each time point and cell size change "acceleration" was calculated as the second derivative (d^2N/dt^2) of the apoptotic cell population in each time point (* depict a significance at the p<0.01 level between concentrations. "**Total**" implies the change rate and change rate acceleration respectively from time point 0 h to 72 h).

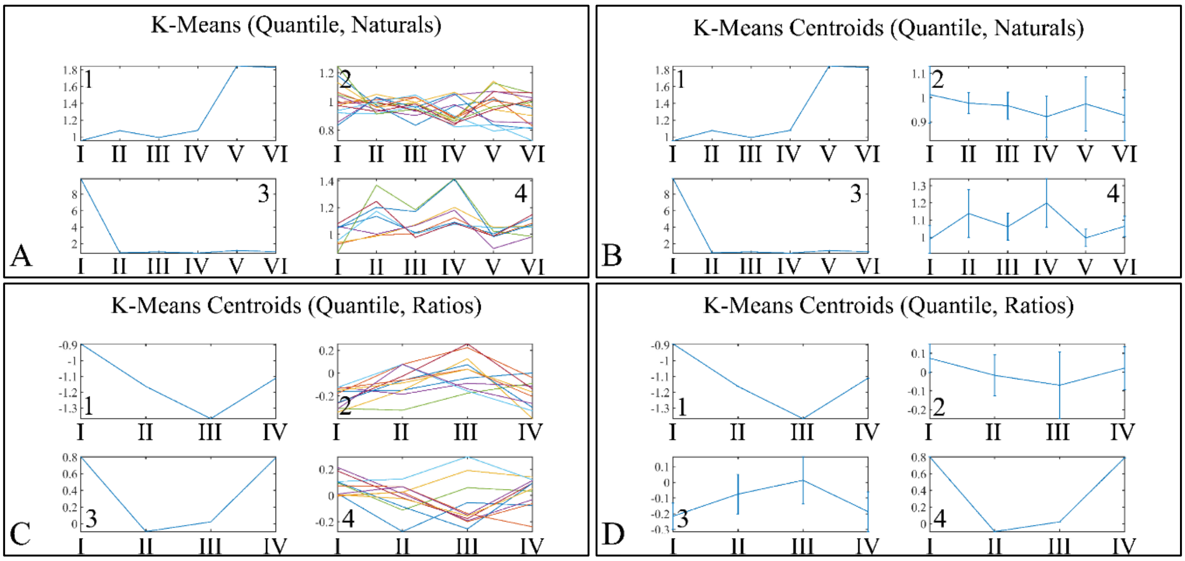

Figure S5. K-means clustering of DEGs. Both natural values (**A**) and their centroids (**B**), as well as gene ratios (**C**) and their respective centroids (**D**) are presented (**Legend**: **I**: 0 μM 4 h, **II**: 0 μM 72 h, **III**: 10 nM 4 h, **IV**: 700 μM 4 h, **V**: 22 μM 72 h, **VI**: 700 μM 72 h, for subfigures **A** and **B**. **I**: 10 nM 4 h, **II**: 700 μM 4 h, **III**: 22 μM 72 h, **IV**: 700 μM 72 h, for subfigures **C** and **D**).
